# Supplementary material for: Coaggregation Occurs between a Piliated Unicellular Cyanobacterium, Thermosynechococcus, and a Filamentous Bacterium, Chloroflexus aggregans
Source: Microorganisms. 2024 Sep 19;12(9):1904. doi: 10.3390/microorganisms12091904 (PMC11434263; doi:10.3390/microorganisms12091904)
Supplement: Supplementary file 1 [file microorganisms-12-01904-s001.zip › microorganisms-3180569-supplementary.pdf]

Supplementary Materials

Coaggregation Occurs between a Piliated Unicellular Cyanobacterium, *Thermosynechococcus*, and a Filamentous Bacterium, *Chloroflexus aggregans*

Megumi Kono and Shin Haruta *

Department of Biological Sciences, Tokyo Metropolitan University, 1-1 Minami-Osawa, Hachioji 192-0397, Tokyo, Japan

* Correspondence: sharuta@tmu.ac.jp; Tel.: +81-42-677-2580

Table S1. DNA sequencing primers to confirm proper segregation of the *pilB* gene in *Thermosynechococcus* sp. NK55a via chloramphenicol resistant gene cassette.

Name	Sequence (5' – 3')
pUC19_upNKpilB_9F	GCGGCCTTTTTACGGTTCC
pUC19_upNKpilB_10R	TGCCGTGGTTGGTCTTTTCC
NKpilB_CmR_11F	TACCGATTGATACCTGCCGC
NKpilB_CmR_12R	GGGTGGGCGTATAGGGAATG
pUC19_dwNKpilB_13F	TGAGGTGCGGATACGAGATG
pUC19_dwNKpilB_14R	CGGGCCTCTTCGCTATTACG
Seq_NKpilB_15F	CCAATGGTGCGAAAAGGAGT
Seq_NKpilB_16R	CTCTGCTGTGCATTGTGCTG
Seq_CmR_17R	AATACGCCCGGTAGTGATCT
Seq_CmR_18F	ATGGCAGAAATTCAGCTTGGCC
Seq_NKpilB_19F	CTATTGCATGACTCCCCGCT
Seq_NKpilB_20R	GGGGTTAATTTACCGCTGATGC

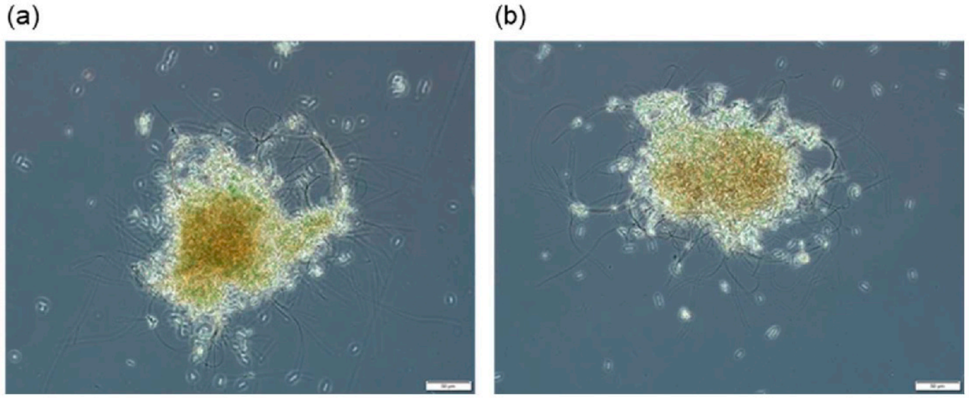

Figure S1. Bright-field microscopic images of cell aggregates after cellulase treatment
Cell aggregates after 24 h incubation were harvested by centrifugation at 10000 × *g* for 1 min, suspended in 50 mM sodium acetate buffer (pH 5.0) with cellulase (1 U/mL, *Trichoderma reesei*) (Worthington Biochemical, Lakewood, NJ, USA) and incubated at 37 °C for 4 h. (**a**) incubated with cellulase; (**b**) incubated without cellulase. Bars, 20 μm.

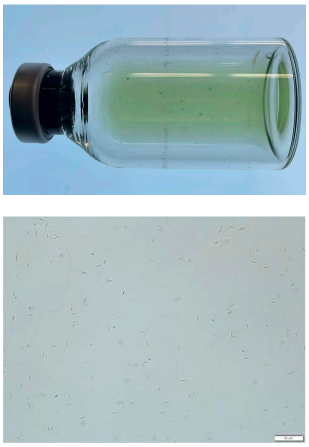

Figure S2. Co-cultivation of *Thermosynechococcus* sp. NK55a and *Chloroflexus aurantiacus* J-10-fl under incandescent light.

C. aurantiacus J-10-fl (=DSM 635 ^{T}) obtained from culture collection was pre-cultivated under the same conditions with that for *C. aggregans* NBF. Cells at the late exponential growth phase were harvested by centrifugation and suspended together into a 5 mL BG11 medium in the same way as the co-cultivation with *C. aggregans* shown in Figure 1. The cell mixture in a 25-mL glass vial was incubated at 50°C in the light (incandescent lamp, 15~20 μmol/s/m^2) using a roller apparatus at 20 rpm (VMRC-5; AS ONE, Osaka, Japan). Photographs of the glass vial (top) and bright field micrographs of the culture solution (bottom; bars, 50 μm) after 12 h incubation.
